# The Crosstalk between IL-22 Signaling and miR-197 in Human Keratinocytes

**DOI:** 10.1371/journal.pone.0107467

**Published:** 2014-09-10

**Authors:** Galya Lerman, Moran Sharon, Raya Leibowitz-Amit, Yechezkel Sidi, Dror Avni

**Affiliations:** 1 Laboratory of Molecular Cell Biology, Center for Cancer Research and Department of Medicine C, Sheba Medical Center, Tel Hashomer, Israel; 2 Institute of Oncology, Sheba Medical Center, Tel Hashomer, Israel; 3 Sackler School of Medicine, Tel Aviv University, Tel Aviv, Israel; IRCCS-Policlinico San Donato, Italy

## Abstract

The interaction between the immune system and epithelial cells is tightly regulated. Aberrations of this balance may result in inflammatory diseases such as psoriasis, inflammatory bowel disease and rheumatoid arthritis. IL-22 is produced by Th17, Th22 and Th1 cells. Putative targets for IL-22 are cells in the skin, kidney, digestive and respiratory systems. The highest expression of IL-22 receptor is found in the skin. IL-22 plays an important role in the pathogenesis of T cell-mediated inflammatory diseases such as psoriasis, inflammatory bowel disease and rheumatoid arthritis. Recently, we found that miR-197 is down regulated in psoriatic lesions. In the present work we show that miR-197 over expression inhibits keratinocytes proliferation induced by IL-22 and keratinocytes migration. In addition, we found that IL-22 activates miR-197 expression through the binding of phosphorylated STAT3 to sequences in the putative promoter of miR-197. Finally we found that IL-22 receptor subunit IL22RA1 is a direct target of miR-197. Hence, we identified a novel feedback loop controlling IL-22 signaling, in which IL-22 induces miR-197, which in turn, negatively regulates IL-22 receptor and attenuates the biological outcome of such signaling. Regulation of this pathway may be important in inflammatory skin disorders such a psoriasis and in wound healing.

## Introduction

Micro-RNAs (miRNAs) are small non-coding RNAs with important roles in post-transcriptional gene expression regulation. More than a hundred miRNAs are expressed in the skin [1]. MiRNAs were found to be essential for skin development in a conditional knock-out mouse model of Dicer, a cardinal enzyme for miRNA processing. Loss of Dicer in keratinocyte (KC) produced several distinct defects in the skin [1], [2].

Our knowledge on the role of miRNAs in skin development stems mainly from studies on miRNA expression in skin disorders. We and others explored the differential expression of miRNAs in normal skin, psoriatic lesions, un-involved skin from psoriatic patients [3], [4]. Others also compared psoriasis patient’s skin to miRNA expression from skin of patients with atopic eczema [5], [6]. Experimental systems of human KC revealed that miRNAs can target major components in KC development. MiR-203 was found to target p63 [7], [8] and miR-125b was found to target KGFR [9]. Both proteins are well known to be involved in skin differentiation and in epithelial repair processes [10], [11]. We found that miR-99a targets IGF-1R [4], a major player in KC proliferation and differentiation [12]. Despite these findings, many functions of miRNAs in skin are still largely unknown and their role in the pathogenesis of skin disorders is even less understood. One of the characteristics of psoriasis is the cross talk between activated immunocytes and KC that begins early upon lesion formation and culminates in the mature psoriatic plaque [13]. Pathogenic T cells, releasing a cascade of cytokines, infiltrate the skin and trigger a hyper-proliferative response of KC [14]. A discrete population of lymphocytes, namely Th17 cells, was significantly more abundant in the psoriatic skin and seems to play a major role in the pathogenesis of psoriasis [15]. Th17 cells depend on IL-23 for their development, survival and proliferation, they produce IL-17A, IL-17F, TNF-α, IL-21 and IL-22 [16], [17].

Th22 cells, which lack the ability to produce IL-17 and IFN-γ, also, produce IL-22. Th22 cells express the chemokine receptor CCR6 and the skin homing receptors CCR4 and CCR10, allowing for infiltration into the skin. They are enriched in the lesional skin of inflammatory skin diseases. This indicates the importance of IL-22 in skin homeostasis and the pathogenesis of skin diseases (review in [18]).

IL-22 is a member of the IL-10 cytokine family which is secreted by activated Th1, Th17 and NK-cells [19]. IL-22 acts via a heterodimeric receptor comprising of IL22RA1 and IL-10RB. It induces the activation of JAK1, Tyk2 and the phosphorylation of STAT1, STAT3 and STAT5 [20]. Cells that express the IL-22 receptor can be found in the skin, kidney, the digestive and respiratory systems [21]. IL-22 plays a major role in the pathogenesis of psoriasis [15], [22]. Psoriatic patients have markedly elevated IL-22 plasma levels, which correlate with disease severity [23]. IL-22 triggered KC hyperplasia in an in vitro reconstituted human epidermis system [24]. Moreover, neutralization of IL-22 prevented the development of psoriasis in a SCID mouse model of the disease [25]. These findings form the experimental basis for the possible utilization of IL-22 pathway inhibitors as therapeutic means in psoriasis [26].

We and others previously showed that miR-197 is significantly down regulated in psoriatic lesions compared to normal skin [4], [27]. We previously showed by qRT- PCR, that the expression of mir-197 was ∼3-fold lower in psoriatic skin as compared to normal skin. This was also confirmed by *In situ* hybridizations [4]. Several studies showed that miR-197 expression is altered in several cancer types [28]–[31] and in type II diabetes [32]. The present study is aimed to investigate its biologic roles in KC and in skin biology.

## Results

### MiR-197 over-expression decreases proliferation and induces differentiation of KC

**Figure 1:**
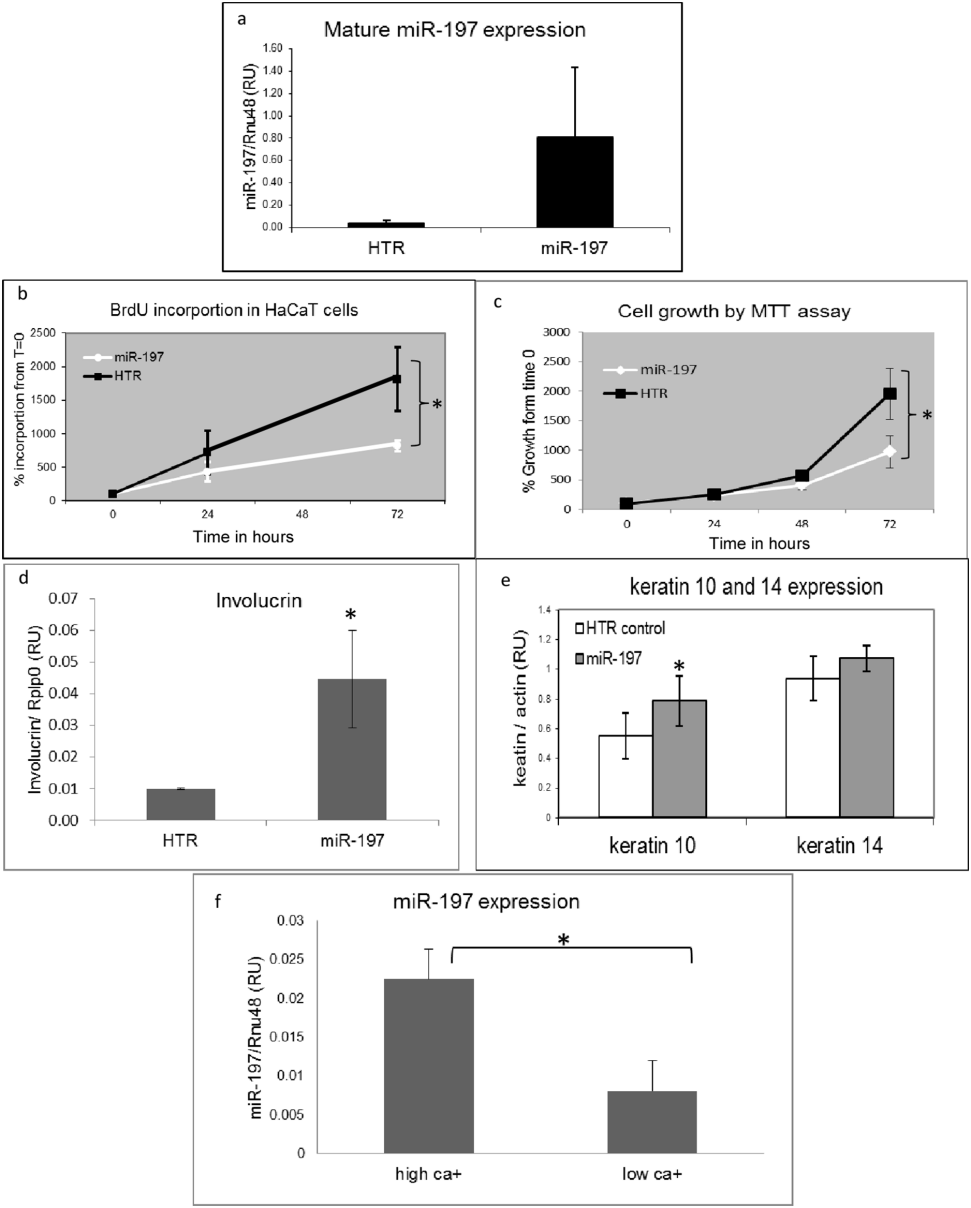
The effect of miR-197 on proliferation and differentiation of KC. a) HaCaT cells were transfected with miR-197 expressing plasmid or HTR expressing plasmid as a control. Total RNA was extracted from each cell line, subjected to qPCR analysis and normalized by Rnu48 (*P<0.049988). b–c) Cell Proliferation of HaCaT cells stably expressing hsa-miR-197 or the HTR control RNA was assessed by (b) BrdU incorporation (c) MTT and assays. Proliferation was calculated as the percentage of the reading at seeding time (T = 0) given a value of 100%. For the BrdU assays mean and standard deviation were calculated from 3 independent experiments (*P<0.0232). For the MTT assays mean and standard deviation were calculated from at list 5 independent experiments (*P>0.002638) as calculated by ttest. d–e). Total RNA of HaCaT-miR-197 or HaCaT-HTR was subjected to qPCR analysis for involucrin, keratin 10 (K10) or keratin 14 (K14) and normalized by Rplp0. Y bars are arbitrary units that define fold change *p = 0.05.f). Total RNA of HaCaT-miR-197 or HaCaT-HTR was subjected to RT-PCR analysis for K10 and K14 and normalized by β-Actin expression. *p = 0.01. f) PHK were seeded in high Ca++ medium or low Ca++ medium. After 24 h cells were harvested and subjected to miR-197 qPCR as in *p = 0.05.

The immortalized KC cell line HaCaT was stably transfected with miR-197 (HaCaT-miR-197) (Fig. 1a). Stably expressing miR-197 HaCaT cells were chosen as a model system, rather than primary human KC (PHK), due to the fact that miRNA mimics are diluted and lost during cell division. Moreover, according to our results the replication rate of cells which absorb the miRNA is diminished, hence, in few hours the non-transfected cells populate the culture. BrdU incorporation was decreased in HaCaT-miR-197 cells relative to HaCaT cells transfected with HTR control RNA (HaCaT-HTR) at both the 24 h and 72 h time points (Fig. 1b). MTT assay confirmed the BrdU assay trend (Fig. 1C). As can be seen in Fig. S1, the apoptotic rate in control or miR-197 over expressing cells over expression of miR-197 does not activate apoptosis in these cells. The expression of involucrin, K10 and K14, KC differentiation markers were evaluated; involucrin and K10 were higher in HaCaT-miR-197 cells vs. control cells while k14 expression did not change (Fig. 1d–e). Furthermore, in PHK that were grown in high Ca^++^ medium, conditions known to drive keratinocytes towards differentiation [33], MiR-197 expression was elevated compared to its expression in cells grown in low Ca^++^ medium (Fig. 1f). These results indicate that miR-197 slows KC proliferation rate and directs them towards differentiation. Our result in psoriatic lesion showed a decrease in the expression of miR-197, therefore we tried to determine the effect of hsa-miR-197 depletion on KC proliferation. A knockdown of miR-197 using antago-mir did not affect the growth or proliferation of HaCaT cells (Figure S2).

### IL-22 enhances the expression of miR-197

**Figure 2:**
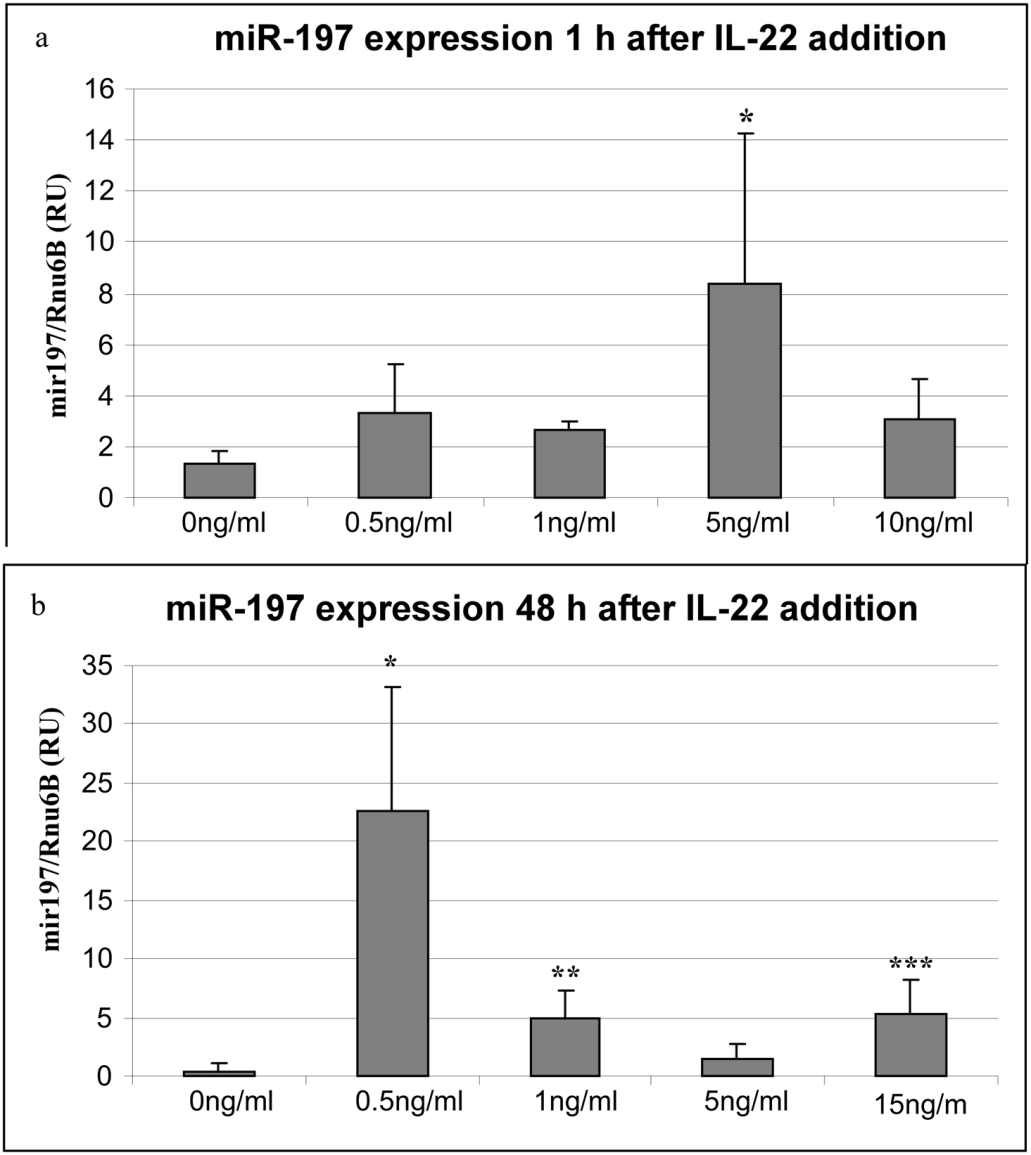
IL-22 enhances miR-197 expression. IL-22 was added to PHK cells at the indicated concentrations. Cells were harvested and subjected to miR-197 specific qPCR. a) 1 h post IL-22 addition *P = 0.022 b) 48 h post IL-22 addition. *p = 0.01 **P = 0.017 ***p = 0.03. (The mean −/+ SD was calculated from 4 independent experiments).

To study the cross-talk between miR-197 and IL-22 pathway, we monitored the expression of miR-197 in primary human KC (PHK) cells treated with different concentrations of IL-22. We performed a dose-response and a time-course to choose the IL-22 concentration and treatment timing. The levels of miR-197 increase significantly in cells treated with 5 ng/ml of IL-22 for 1 h or 0.5 ng/ml IL-22 for 48 h as compared to untreated cells (Fig. 2a–b). These results indicate that IL-22 signaling enhances the expression of miR-197.

### STAT3 mediates between IL-22 and miR-197 expression

**Figure 3:**
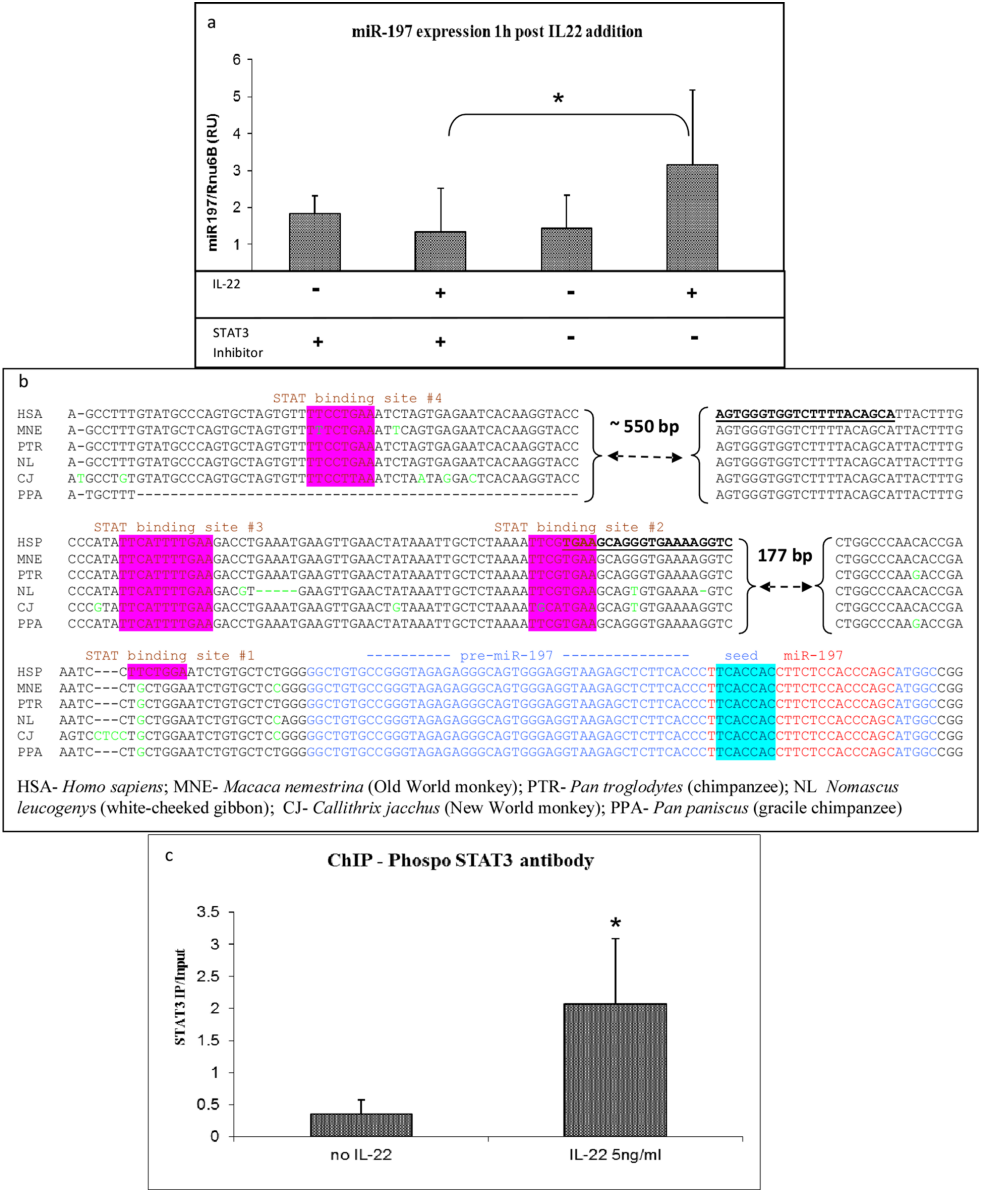
STAT3 binds to the putative promoter region of miR-197 after IL-22 treatment. a) STAT3 inhibitor (final concentration of 100 µM) was added to PHK, 30 min after 5 ng/ml IL-22 was added. a) 1 h afterword miR-197 quantity was measure by qPCR (The mean −/+ SD was calculated from 4 independent experiments) *p = 0.047699) b) Alignment of genomic miR-197 gene region sequence in six Primates. Pri-miR-197 marked in blue; mature miR-197 marked in red; miRNA “seed” marked in blue box. Different bases form consensuses are in green. The putative promoter region has 3 conserved STAT sites and one un-conserved (pink boxes). c) PHK cells were treated or not with 5 ng/ml IL-22 for 30 min, then were subject to ChIP assay using phosphorylated STAT-3 antibody. The results present the amount measured by PCR of immune precipitated DNA with the anti pSTAT3 divided to the amount of measured by PCR of input DNA. All PCR were done with qPCR SYBR Green dye. The mean −/+ SD was calculated from 4 independent experiments (t test *P = 0.016). Primers that were used in the ChIP assay are marked by underline in b.

Similar to other members of the IL-10 cytokine family, IL-22 utilizes JAK/STAT signaling, predominantly through activation of STAT3 [26]. To study whether miR-197 expression is directly regulated by the IL-22-STAT3 pathway, PHK were treated with a specific STAT3 inhibitor, S3I-201 [34]. S3I-201 prevents the IL-22-induced expression of miR-197 (Fig. 3a). These results indicate that the increase in miR-197 expression in response to IL-22 is mediated through STAT3.

STAT3 is known to undergo phosphorylation and dimerization following IL-22 signaling, after which it binds to promoters and activates transcription of target genes [35]. We therefore asked whether the putative promoter of miR-197 is a target of activated STAT3. The miR-197 gene is located on human chromosome 1p13.3, in a region distinct from other known transcription units. The bioinformatics tools Tfsitescan [36] and TFSEARCH [37] mapped four putative STAT binding sites (TTN_(4–6)_AA) [38] within the ∼2000 bases upstream of the miR-197 gene, its potential promoter (Fig. 3b). The sites designated #2, #3 and #4 are highly conserved among primates, suggesting that they are of evolutionary significance. Using chromatin immunoprecipitation (ChIP) assay we examined whether activated p-STAT3 binds to the putative miR-197 promoter sites following IL-22 treatment. The addition of IL-22 resulted in significant enrichment of precipitation of the miR-197 putative promoter area by p-STAT3 antibodies (Fig. 3c). These results indicate that upon treatment of PHK by IL-22, activated STAT3 binds to the putative miR-197 promoter region.

### The IL22RA1 subunit is a direct target of miR-197

**Figure 4:**
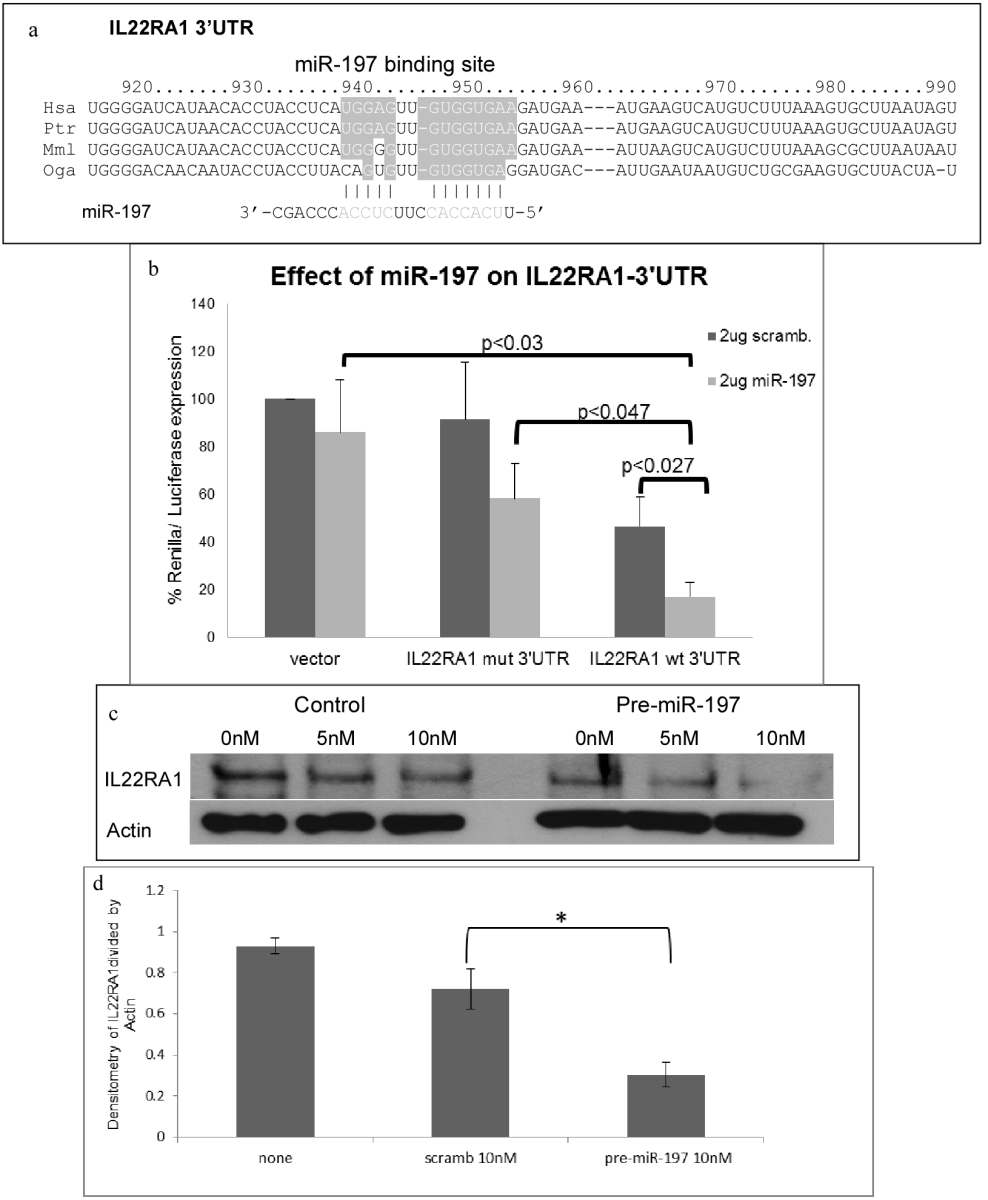
miR-197 suppresses the expression of IL22RA1 by binding to its 3′UTR. a) miR-197 binding site on IL22RA1 3′UTR. b) HEK-293T cells were transfected with psi-CHECK2 vectors encoding luciferase (vector), luciferase fused to the IL22RA1-3′UTR (IL22RA1 wt 3′UTR), or luciferase fused to a mutated in miR-197 binding to the IL22RA1 3′UTR (IL22RA1 mut 3′UTR) together with 2 µg miR-197 expressing plasmid or 2 µg scramble RNA expressing plasmid. Cells transfected with only with vector lacking the IL22RA1 3′UTR was valued as 100%. The error bars are calculated as standard error of at least 6 independent experiments. c) Western blot (WB) analysis of IL22RA1 protein 48 h after transfection with 5/10 nM of scramble control pre-miR or with 5/10 nM of pre-miR-197. d) Densitometry analysis of 4 WBs analysis of IL22RA1 protein 48 h after transfection with 10 nM of scramble control RNA (scramb), or 10 nM pre-miR-197 *p = 0.0000818.

IL-22 exerts its effects through a heterodimeric receptor complex consisting of IL22RA1 and IL-10RB. Bioinformatics analysis using the Web-based tool ‘target scan’ (www.targetscan.org) revealed that both subunits are potential targets of miR-197. Putative interactions of miR-197 with the IL22RA1 3′UTR and IL-10RB 3′UTR are shown in Fig. 4a and Fig. S3a. To determine whether IL22RA1 or IL10RB are miR-197 targets, cells were co-transfected with a plasmid containing the IL22RA1 3′UTR or the IL-10RB 3′UTR downstream of the luciferase reporter together with a miR-197 expressing plasmid, and luciferase reporter assay was performed 72 h later. Luciferase expression was significantly lower in cells transfected with the luciferase-IL22RA1-3′UTR plasmid together with miR-197 expressing plasmid than in cells transfected with a plasmid lacking the 3′UTR of IL22RA1 (Fig. 4b). Moreover, there is a significant difference (p = 0.027) when comparing the luciferase active of luciferase-IL22RA1-3′UTR plasmid in the presence or absence of miR-197. To further explore miR-197 effect on IL22RA1, we generated a IL22RA1-3′UTR-luc mutant in which four nucleotides in the seed response sequence were changed from GUGGUGAA to GUaacaAA. The mutant was co-transfected with the miR-197 expression plasmid as before and luciferase activity was assessed. Figure 4b (IL22RA1 mutant 3′UTR panel) clearly demonstrates that miR-197 has lesser effect on the mutated IL22RA1-3′UTR; there is no difference between the luciferase active in the presence or absence of miR-197 (p = 0.2199). All proving that miR-197 seed sequence at the IL22RA1 3′UTR is essential for the regulation of IL22RA1 by miR-197.

The same results we observed with 5 nM of pre-miR-197 RNA, as seen in figure S2. This miR-197 repression was released in cells transfected with pre-miR-197 RNA together with antago-miR-197, this also suggests that the antago miR-197 is active (Figure S3).

In contrast, the other subunit of the receptor to IL-22, IL10RB, seems not to be a target of miR-197, as indicated by the luciferase reporter assays with the IL10RB 3′UTR and miR-197 (Figure S4).

The effect of miR-197 on IL22RA1 expression was further examined by Western blot (WB) analysis. Over expression of pre-miR-197 in PHK cells led to a dramatic decrease in the level of IL22RA1 protein (Fig. 4c–d). These results, taken together, indicate that IL22RA1 and not IL-10RB is a direct biochemical target of miR-197.

### Mir-197 inhibits the effects of IL-22 on KC phenotypes

**Figure 5:**
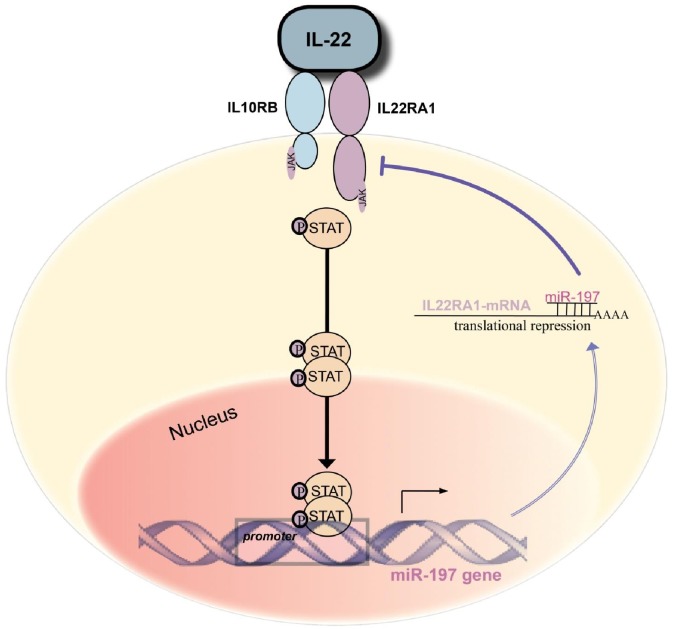
Feedback loop model between IL-22 signaling and miR-197.

**Figure 6:**
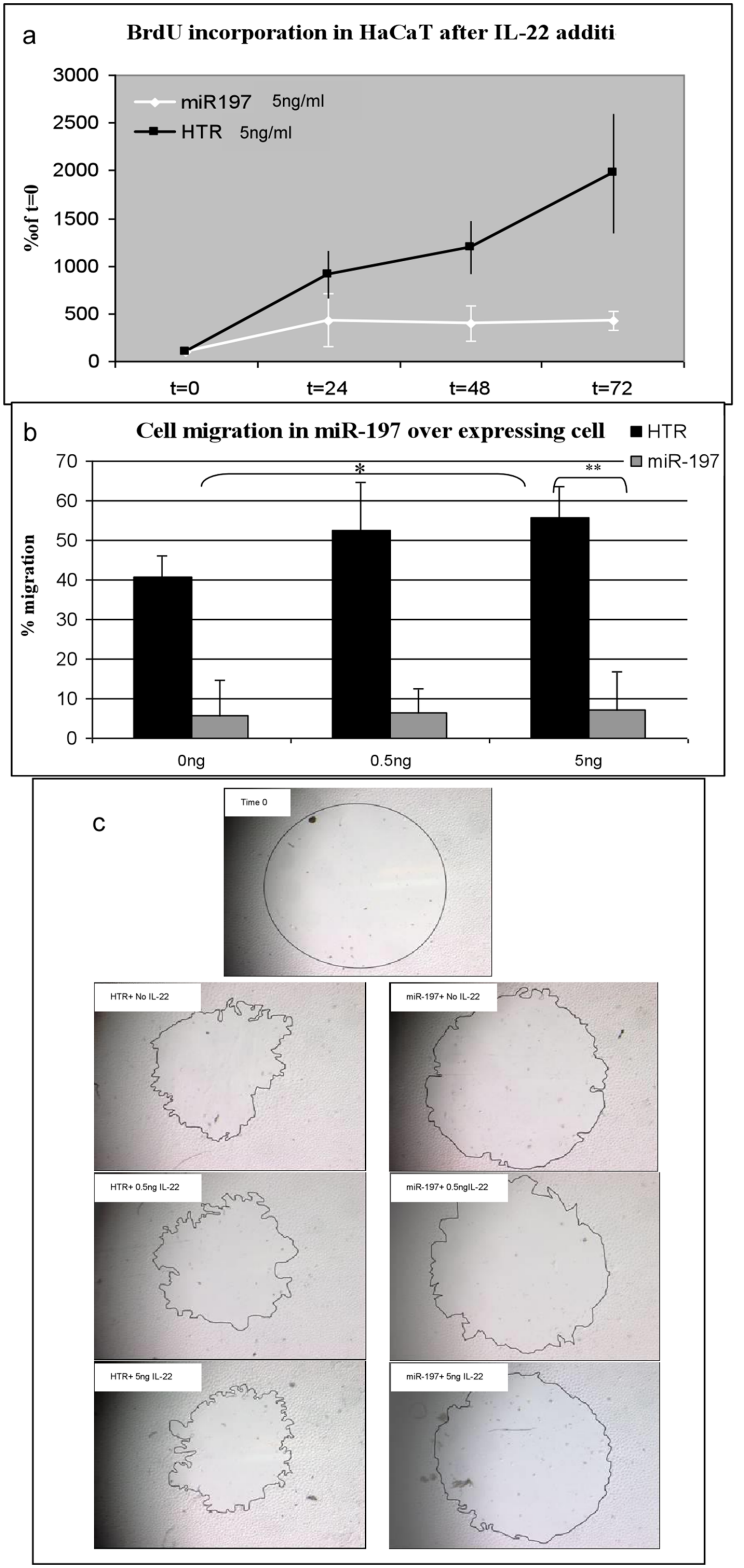
miR-197 slows proliferation and migration of KC. HaCaT-miR-197 or control HaCaT–HTR were subject to; a) BrdU incorporation as in figure 1a. 24 h after seeding, at the 0 time point, 5 ng/ml IL-22 was added. Cell Proliferation was calculated as in figure 1a. Mean and standard deviation were calculated from 3 independent experiments (*P<0.0145). b) 24,000 cells were seeded on platypus 96 wells plate to reach 80% confluence. Then cells were serum starved for 24 h, afterward, IL-22 was added as written. 48 h later, cells were washed and fixated. C) Representative experiment of Cell migration: percentage was calculated as the subtraction of the area, as marked, from the area at time 0. Area was determined by imageJ program. (p<0.001 **p<0.000001).

Our results reveal that IL22RA1 is regulated by miR-197; moreover, they strongly suggest that IL-22, activates the transcription of miR-197 through STAT3 signaling, thus generating a biochemical feedback loop as summarized in Fig. 5. It was previously shown that IL-22 enhances KC proliferation, increases the thickness of reconstituted human epidermis, inhibits KC differentiation [24], [39], and enhances KC migration [40], [41]. We asked whether these biological effects of IL-22 will be affected by miR-197 over-expression. To address this question we measured the BrdU incorporation in IL-22 treated HaCaT-miR-197 or HaCaT-HTR, and found it to be significantly higher in the HaCaT-HTR cells (Fig. 6a). In order to evaluate the effect of miR-197 over-expression on IL-22-induced cellular migration, we conducted an in vitro cell migration assay in HaCaT-miR-197 vs. HaCaT-HTR. After seeding, cells were serum starved for 24 h, next IL-22 was added for additional 48 h and cells were fixed. The control HTR-HaCaT migrated to cover 40% of the empty area. The addition of 0.5 or 5 ng/ml IL-22 in the serum free medium led to an increase in the covered area of 52% and 56%, respectively, signifying IL-22-iduced motility. HaCaT-miR-197 had a significantly lower level of baseline migration, covering only 5–10% of the empty area at 48 h, without any significant change in migration following IL-22 treatment (Fig. 6b).

In parallel we treated HaCAT with antago-miR-197, in the presence or absence of IL-22. Our results suggest that the antago-miR-197 had no significant effect on KC proliferation (figure S5 a–b).

The miRNAs role as “fine-tuner” might explain the fact that in high over expression of repressor miRNA we were able to induce biological effects, however down regulation of one out of many “fine tuners” had no biological effect.

### DNA methylation of the miR-197 putative promoter regions

We previously showed that miR-197 expression is significantly decreased in psoriatic lesions [4]. Our current results suggest the existence of a novel biochemical inhibition loop; IL-22, as previously shown, through IL22RA1-IL10RB, activates STAT3 which then enhanced the expression of miR-197, and miR-197 targets the expression of IL22RA1, thereby closing the biochemical feedback loop. However, despite the high levels of IL-22 in the blood of psoriatic patients and high expression of IL22RA1 in psoriatic patients’ skin, the expression of miR-197 is decreased in their KC.

**Figure 7:**
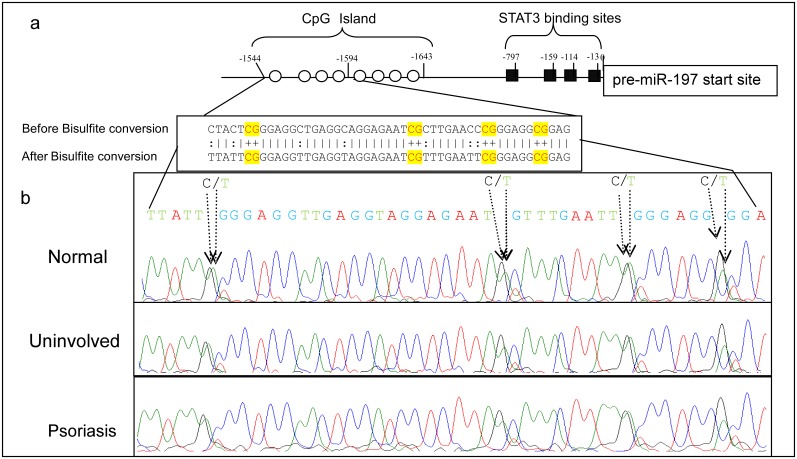
Cytosine methylation in psoriatic and normal skin. a) Schematic presentation of miR-197 putative promoter. The 8 circles represent the CpG, of the CpG Island. Sequence from −1544 to −1594 before or after bisulfite conversion is shown. b) Represented sequencing chromatogram of the bisulfite conversion region, analysis by BioEdit, C and T residues at the CpG are marked.

Recent work suggest that DNA methylation in the skin is dynamic and it changes along the epidermal layers and in specific genes [42]. DNA methylation is capable of regulating both gene repression and activation, and the basal status of promoter methylation is important for individual genes expression [43], [44]. A recent study comparing differences in the DNA methylation, between psoriatic lesions and uninvolved or normal skin revealed many CpG sites with differential methylation levels [45]. We hypothesized that cytosine methylation of the miR-197 putative promoter in psoriatic lesion compare to normal skin might be different and thereby explain why miR-197 is silenced in psoriasis despite the high levels of IL-22 in the patients’ blood. The ∼2000 bases up stream of the pre-miR-197 sequences comprise the regulatory elements of promoter and contain one CpG island (Figure 7a). We analyzed the DNA methylation of this region in biopsies from formalin fixed paraffin embedded (FFPE), of psoriatic lesions, uninvolved psoriatic skin and normal skin. Each sample was subjected to at least two sequencing analyses. We can see that the methylation pattern of the mapped CpG island in the miR-197 putative promoter, in psoriatic samples is similar to normal skin (figure 7b).

## Discussion

Recently we have shown that miR-197 expression is down regulated in the psoriatic lesions compared to normal or uninvolved psoriatic skin [4]. We now show that miR-197 leads to decreased proliferation and migration of KC in vitro, along with increased expression of KC differentiation marker, involucrin and keratin 10. It is noteworthy that despite the observed inhibition of proliferation of HaCaT cells stably over expressing miR-197, exposure of HaCaT cells to antago-miR-197 did not affect cell proliferation. The experiment shown in figure S3 and discussed in the results, proves that the antago-miR-197 is indeed active, since it releases miR-197 effect on reporter mRNA containing the 3′UTR of IL22RA1 (figure S3). The lack of antago-miR effect on proliferation suggests that in order to inhibit proliferation it is sufficient to over express, in large excess, one miRNA. However, in order to unbalance this very complex, multifactorial system and induce hyper proliferation phenotype, it is possible that the partial of only one of these components is not sufficient.

In this study, we establish that the IL-22 receptor subunit IL22RA1 is a target of miR-197 (Fig. 4, and figure S1C). Interestingly, treatment of cells with IL-22 increases the expression of miR-197 (Fig. 2). Bioinformatics predict that miR-197 targets the two units of the hetrodimeric IL-22 receptor, namely IL22RA1 and IL-10RB. Our work shows that only IL22RA1, but not IL-10RB, is a bona-fide target of miR-197 (Fig. 4, S1). IL22RA1 subunit is part of the receptor of both IL-22 and IL-20 [19]. Both cytokines are up regulated in psoriasis and have similar biological effects [46]. While IL-22 utilizes only the IL22RA1/IL-10RB receptor, IL-20 can utilize also the IL-20RA1/IL-20RA2 receptor [19]. However, in psoriasis, only the IL22RA1 subunit is up regulated [47]. This implies that miR-197, by controlling IL22RA1, can fine-tune several aspects of cytokine signaling pathways. Moreover, the fact that miR-197 targets only the IL22RA1 subunit of the IL-22 receptor emphasizes the role it might play in the pathogenesis of psoriasis.

**Figure 8:**
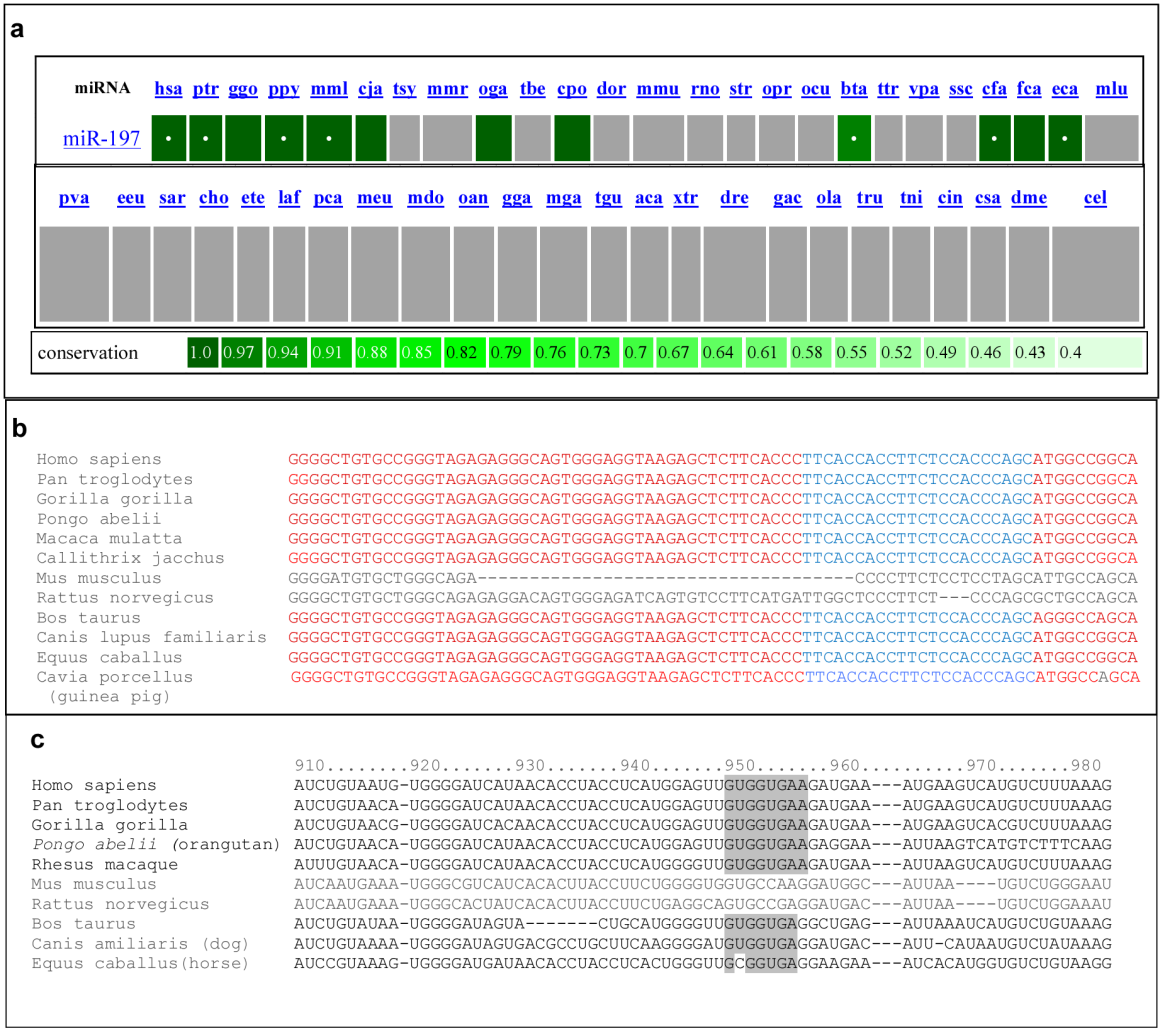
Evolutionary conservation of miR-197 and its putative binding site on IL22RA13′UTR. a) Using the miRviewer program, the present of miR-197 was examined in various animals genome. The name of the species is written above each block. The white dot in box indicates that this miRNA are present in miRbase. Grey box indicates that the miRNA was not identified in the genome of the specific species, under stringent parameters. The tone of green defined the degree of conservation b) Alignment of pre-miR-197 gene sequences of few primates, mouse, rat, cow, dog, horse, and guinea pig. c) Alignment of the IL22RA1 3′UTR of some of the above species and the miR-197 putative binding site is marked.

There is no mouse homolog of miR-197. The miRviewer program [48] reveals that the miR-197 gene exists only in some placental mammals (primates, horse, cow, dog, cat and guinea pig; Fig. 8 a,b). According to the miRBase data, miR-197 gene is even more infrequent, being present only in primates, horse, cow and dog [49]. IL-22 receptor gene is expressed in non-mammals as well as in mammals. Yet the binding site of miR-197 in the 3′UTR of IL22RA1 is found only in primates, dog, cow and horse but not in mouse or rat Figure 8C. Interestingly, there are no records of spontaneous development of psoriasis in mouse. Apart from humans, only two types of monkeys are reported to develop a type of psoriatic plaques [50], [51], and dogs are also reported to develop lichenoid-psoriasiform dermatosis resembling psoriasis [39], [52]. It is tempting to speculate that co-evolution of the miR-197 gene and the IL22RA1 subunits occurred in human and some other mammals, and that abnormal function of this interaction contributes to the pathological development of psoriatic skin disease in these species.

In this manuscript we focused on IL22RA1 as target of miR-197. However, the biological effects we have observed in keratinocytes overexpressing miR-197; inhibition of proliferation and differentiation (figure 1) probably are the result of additional, more complex effects of miR-197 on additional targets. This issue will be dealt in future studies.

Proteins from the STAT family and miRNAs were suggested to form complex networks (reviewed in [53]). Like other members of the IL-10 family, IL-22 primarily activates the JAK/STAT pathway following IL-22 receptor activation (reviewed in [19]). We show that miR-197 activation by IL-22 is mediated through STAT3 (Fig. 3). Following IL-22 binding to its receptor, the STAT dimers translocate to the nucleus, where they bind to promoters and regulate the expression of target genes (reviewed in [38]). We were able to show by ChIP assay that one of its targets is the miR-197 putative promoter region (Fig. 3c).

Our results show that miR-197 is activated by IL-22 at a specific range of concentrations. Small changes in IL-22 concentration have a dramatic effect on the expression levels of miR-197 and thereby on the levels of IL22RA1 protein. It was recently suggested that the mode of regulation of miRNAs is to establish a threshold level of target mRNA above which protein production is highly repressed. Near this threshold, protein translation responds sensitively to changes in input. This may suggest that miRNAs can act both as a ‘switch’ and as a ‘fine-tuner’ of gene expression. This model might be true for IL-22 signaling, known to be important for wound healing [24]. This process, which involves hyper proliferation and migration of KC, must be under strict regulation, leading ultimately to its gradual halt. It is tempting to speculate that miR-197 has a role in imposing such tight and delicate regulation.

Despite the high output of the IL-22 signaling pathway, miR-197 is down regulated in psoriasis [4]. Similarly, miR-99a is down-regulated in psoriatic KC despite the high output of the IGF1 signaling pathway in psoriasis [4]. Both these observations suggest that aberrant silencing of miRNAs takes part in the pathogenesis of psoriasis by allowing the un-controlled signaling of IL-22 and IGF1R, respectively. This silencing may be the result of epigenetic suppression of the specific gene. This hypothesis is supported by the study of the effect of TNFα on the epigenetic markers of KC [45]. There is growing evidence that epigenetic factors play a role in the pathogenesis of psoriasis. One of them is the fact that the likelihood that both monozygotic twins will have psoriasis is only 35% to 72%, [54]. The knowledge on the epigenetic phenomena consisting of DNA methylation and histone modification and their effect on genes expression is becoming more and more understandable [43], [55]. There are few reviews correlating epigenetics and psoriasis [56]–[58]. Gervin K et. al. compare the whole genome DNA methylation between monozygotic twins, only one of them had psoriasis. They found that in CD4^+^ cells, in a combined analysis of DNA methylation and gene expression, a significant deference of methylation of specific genes known to be involved in immune response and associated with psoriasis [59]. Zhang P. et. al. compared cytosine methylation between psoriasis patients to normal healthy humans. They observed a significant increase in global DNA methylation level in psoriatic peripheral blood mononuclear cells (PBMCs) relative to normal controls. Furthermore, using immunohistochemistry they found extensive DNA methylation staining in skin lesion biopsies from psoriatic patients compared to normal controls. Moreover, they found a significant positive correlation between methylation staining in the skin and Psoriasis Area and Severity Index (PASI) scores among psoriatic patients [60]. The same group showed that global histone H4 hypoacetylation was observed in PBMCs from psoriasis vulgaris patients [61].

In the present study we did not detect any difference in cytosine methylation of the putative miR-197 promoter from biopsies of normal, uninvolved, or psoriatic skins. These results are consistent with the results of Zhang P. et al. (personal communication with Zhang P. [62]).

The fact that there is no change in the methylation of this region between psoriatic and normal skin could be either due to lack of correlation between this CpG island methylation and the expression of miR-197 or due to other epigenetic mechanisms being responsible to this silencing in psoriasis.

This finding may form the basis for in-vivo investigation the epigenetic effects of IL-22 and additional cytokines on promoters of miRNAs known to be silenced in psoriasis.

In summary, this study revel the feedback loop between IL22 and miR-197 and suggests that miR-197 acts as a modulator, linking between the immune system and KC, which may play a substantial role in the pathogenesis of psoriasis. The regulation of this signaling pathway may be important in inflammatory skin disorders and in wound healing.

## Materials & Methods

The research involved with human skin tissue has been approved by the Sheba medical center Institutional Review Board (IRB) committee according to the principles expressed in the Declaration of Helsinki. The approved application number is SMC-9776-12.

### Cells cultures

293T, HaCaT, and PHK were grown as previously described [4], [63]. In some experiments we have chosen HaCaT immortalized KC rather than primary human KC (PHK), due to the fact that miRNA mimics and antagomir are diluted and lost during cell division.

### Cell proliferation by -BrdU incorporation or MTT

Was conducted using Cell Proliferation ELISA, BrdU (colorimetric) kit from (Roche) as previously described [4].

Cell growth was assessed by seeding 3000 HaCaT cells per well in 96-well plates. Viable cell counts were monitored from seeding time (t = 0) to 72 h. Cell counts were determined using the MTT (3-[4, 5-dimethylthiazol-2-yl]-2, 5-diphenyl tetrazolium bromide)-based Cell Growth Determination Kit TOX-1 (Sigma-Aldrich, Israel Ltd. Rehovot 76100 ISRAEL).

### Apoptosis

Apoptosis was evaluated with flow cytometry assay using Annexin V-FITC Apoptosis detection Kit from (eBioscience San Diego, CA) Catalog Number: 88-8005, and was performed on 5×10^5^ cells according to the manufacture protocol.

### Quantitative real time PCR (qPCR)

Total RNA of cells was extracted using Norgen total RNA purification Kit (Norgenbioteccorp #17200). Quantification of miRNA was performed as previously described [4].

### Plasmids

The plasmids pMSCV-miR-197 and pMSCV-HTR were kindly provided by Agami, R. [64]. Luciferase-IL22RA-3′UTR (HmiT016091-MT01), Luciferase-IL10RB-3′UTR (HmiT009687-MT01), and the control no-3′UTR (CmiT000001-MT01) plasmids were purchased from GeneCopoeia, (GeneCopoeiaInc, Rockville, MD 20850USA). The WT psiCHECK-Luciferase-IL22RA-3′UTR was generated by amplifying by PCR reaction fragment of ∼1000 bp containing the 3′UTR of IL22RA1 mRNA from the HmiT016091-MT01 plasmid. The primers used are the following primers (table 1) (marked is the restriction enzyme site). The fragment was cut with XhoI and NotI and ligated into psiCHECK-2 that was cut with the same enzymes.

**Table 1:** Primer used to generate psiCHECK-IL22RA1-3′UTR WT and mutant.

Forward primer with XhoI	5′- CCG***CTCGAG***CGGGGAATGGGAAAGGCTTGGTGC-3′
Reverse primer with NotI	5′- ATAGTTTA***GCGGCCGC***ATTCTTATGCTACCGTTTATTGGGCACTG-3′
Mutant primer	5′- CTCATGGAGTTGT***AACA***AAGATGAAATG-3′

The IL22RA1-3′UTR mutant for the hsa-miR-197 seed sequence was created using the Megaprimer Mutagenesis assay [65] using primer forward and mutant primer (mutant is marketed) (table 1) for the first amplification generated a fragment of ∼100 bp that was used for the second amplification with the reverse primer. The ∼1000 fragment was cut with XhoI and NotI and ligated into psiCHECK-2 that was cut with the same enzymes.

### Transfections

293T and HaCaT were transfected as previously described [4]. PHK cells were transfected using FugeneHD or X-tremeGENE Transfection Reagent.

(Roche,CH-4070, Basel, Switzerland). Stably transfected HaCaT cells were generated by transfecting with plasmids pMSCV-miR-197 or pMSCV-HTR [64] and lines were achieved after selection for 4 weeks with Blasticidin at a final concentration of 16 µg/ml.

### Luciferase Assay

Luciferase assayswereperformed using the Dual-Luciferase Reporter (DLR) Assay System (Promega Corporation Madison, WI 53711 USA), or with Luc-Pair miR Luciferase Assay (GeneCopoeia Rockville, MD 20850 USA).

### Determination of proteins pxpression by WB

WB was performed as described previously [63] Using monoclonal Mouse IgG1 Clone #305405,anti-Human IL22RA1 antibody (R&D Systems, Inc. MN 554193 USA) and β-Actin AC-15 antibody (ab276) (abcam Cambridge, CB4 OFW, UK).

### Chromatin Immunoprecipitation (ChIP) Assay

Was performed as modified protocol from previously described in [66] (See Methods S1).

### Migration Assay

Was performed using Oris Cell Migration Assay (Platypus Technologies Madison WI 53711 USA) (see detailed in Methods S2). Microscope model: OlympusSZX16 Research Stereomicroscope. Olympus SDF PLAPO objective lenses extra-wide zoom range of 7.0x–115x. Camera model: Nikon DSD-Fi1. Acquisition software: AnalySIS getIT. Image processing software: Image-J program.

### Methylation Assay

DNA was extracted using the AllPrep DNA/RNA FFPE Kit (USA QIAGEN Inc.27220, CA 91355) for biopsies or by Archive Pure DNA Cell/Tissue Kits (5 PRIME, Inc.) for PHK. Bisulfate reactions were done with EZ DNA Methylation-Gold Kit (ZYMO research). PCR and sequencing was performed with the specific primers:

F5′: TTTTATTAAAAATATAAAAATTAGTTAGGTATGGT.

R5′: ATAGAGTGAGTTTGTTTTTTTTTTGTT.

The sequencing was analyzed by BioEdit [67].

### Materials

Recombinant Human IL-22 cytokine was purchase from (PeproTech, Rocky Hill, NJ). STAT3 inhibitor (VI, S3I-201) was purchased from form Santa Cruz biotechnology, Inc, city, state. Anago-miR-197 (Anti-miR miRNA Inhibitors, 5 nmol ID AM10354) was purchased from Applied Biosystems).

### Statistical analysis

Statistical significance was done using the Student’s t-test. For a single comparison, a p-value<0.05 was considered significant.

## Supporting Information

Figure S1 **miR-197 over expression does not elevate apoptosis rate in cells.** Effect of HaCaT cells transfected with miR-197 expressing plasmid or HTR expressing plasmid as a control, were subjected to stained with annexin V-FITC as apoptosis marker and PI analysis and where assay by flow cytometry a) percentage annexin stained cells average of three independent experiments b) Represented experiment flow cytometry output.
(TIF)

Figure S2 **Effect of antago-miR-197 on KC proliferation.** HaCaT cells were transfected with antago-miR-197 or scrambled control RNA. Next, BrdU incorporation assay was performed as described in figure 1b.
(TIF)

Figure S3 **Antago to miR-197 releases miR-197 inhibition impose on IL22RA1-3′UTR depended.** HaCaT cells were co-transfected with vector plasmid or luciferase-IL22RA1-3′UTR plasmid and 5 nM of scrambled control RNA, or mimic miR-197 RNA. In parallel one set of cells was transfected with 5 nM of mimic miR-197 together with antago-miR-197. The graph represent and average of at list three independent experiments.
(TIF)

Figure S4 **IL10RB subunit is not a target of miR-197.** A) MiR-197 biding site in the IL10RB 3′UTR. B) HaCaT cells were co-transfected with vector or IL10RB-3′UTR plasmid with a miR-197 expressing plasmid at different concentrations. In each experiment the same set of plasmids were transfected in triplicates. The graph presents the average of 4 independent experiments. The results of cells transfected with vector lacking the IL10RB-3′UTR and without miR-197 expressing plasmid was valued as 100%.
(TIF)

Figure S5 **Effect of antago-miR-197 on KC proliferation.** HaCaT cells were transfected with antago-miR-197 or scrambled control RNA. 24 h later cells were treated or not with the indicated IL-22 concentrations. Next, BrdU incorporation assay was performed as described in figure 1b.
(TIF)

Methods S1 **Chromatin Immunoprecipitation (ChIP) Assay.**
(DOCX)

Methods S2 **Migration Assay.**
(DOCX)
