# Supplementary figures and images for: The Crosstalk between IL-22 Signaling and miR-197 in Human Keratinocytes
Source: PLoS One. 2014 Sep 10;9(9):e107467. doi: 10.1371/journal.pone.0107467 (PMC4160297; doi:10.1371/journal.pone.0107467)

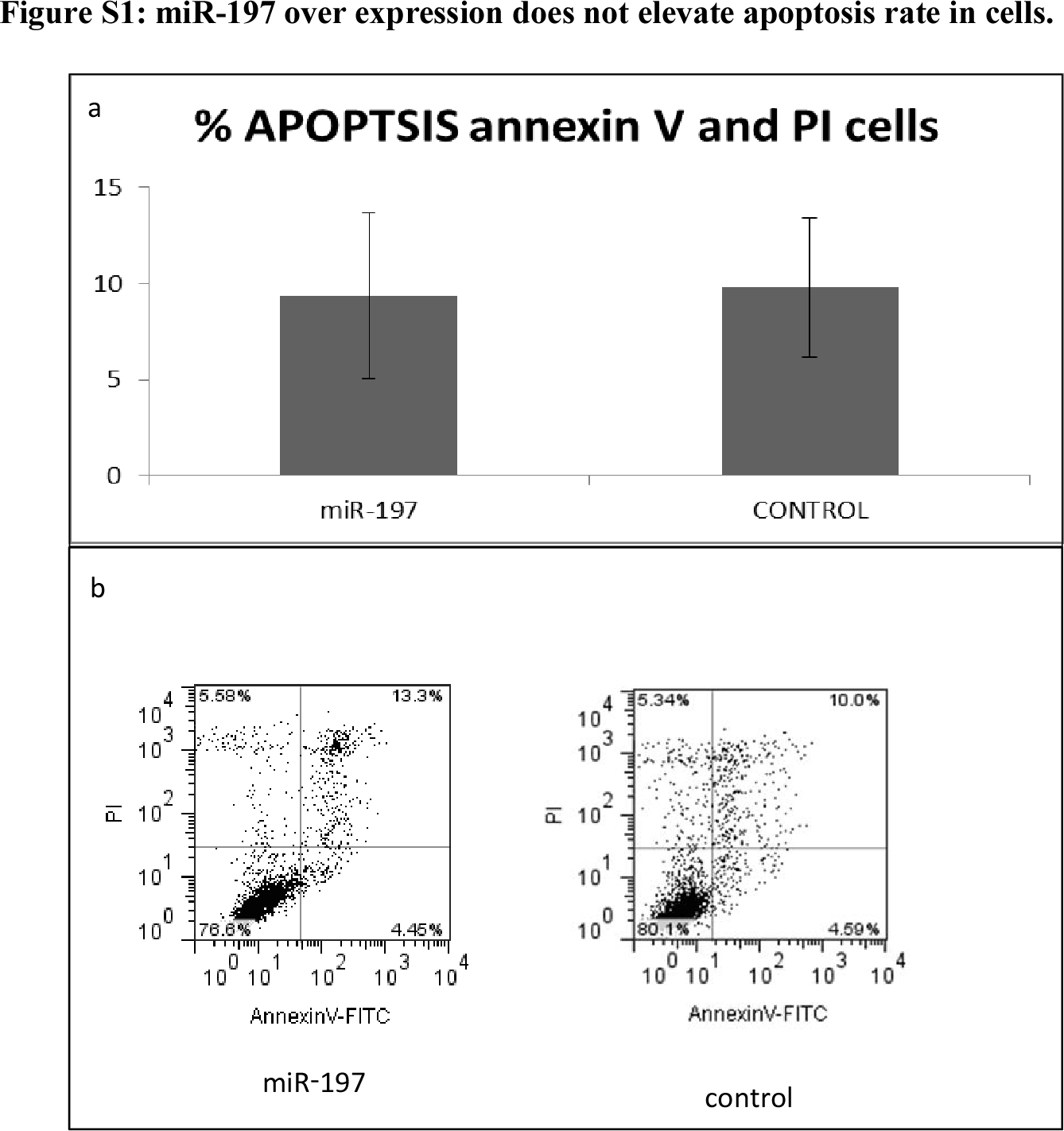

Supplement: Figure S1 — miR-197 over expression does not elevate apoptosis rate in cells. Effect of HaCaT cells transfected with miR-197 expressing plasmid or HTR expressing plasmid as a control, were subjected to stained with annexin V-FITC as apoptosis marker and PI analysis and where assay by flow cytometry a) percentage annexin stained cells average of three independent experiments b) Represented experiment flow cytometry output. (TIF) [file pone.0107467.s001.tif]

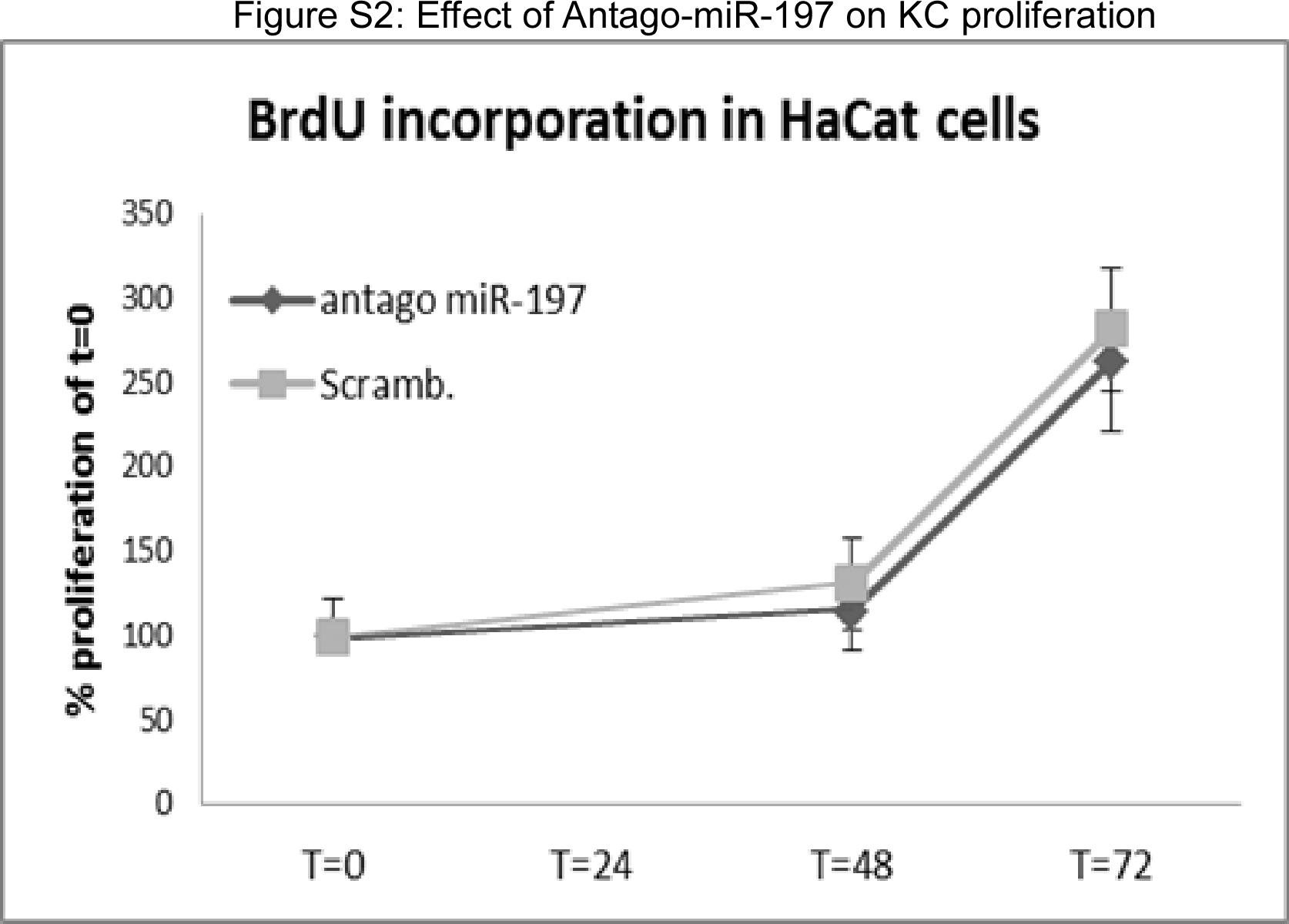

Supplement: Figure S2 — Effect of antago-miR-197 on KC proliferation. HaCaT cells were transfected with antago-miR-197 or scrambled control RNA. Next, BrdU incorporation assay was performed as described in figure 1b. (TIF) [file pone.0107467.s002.tif]

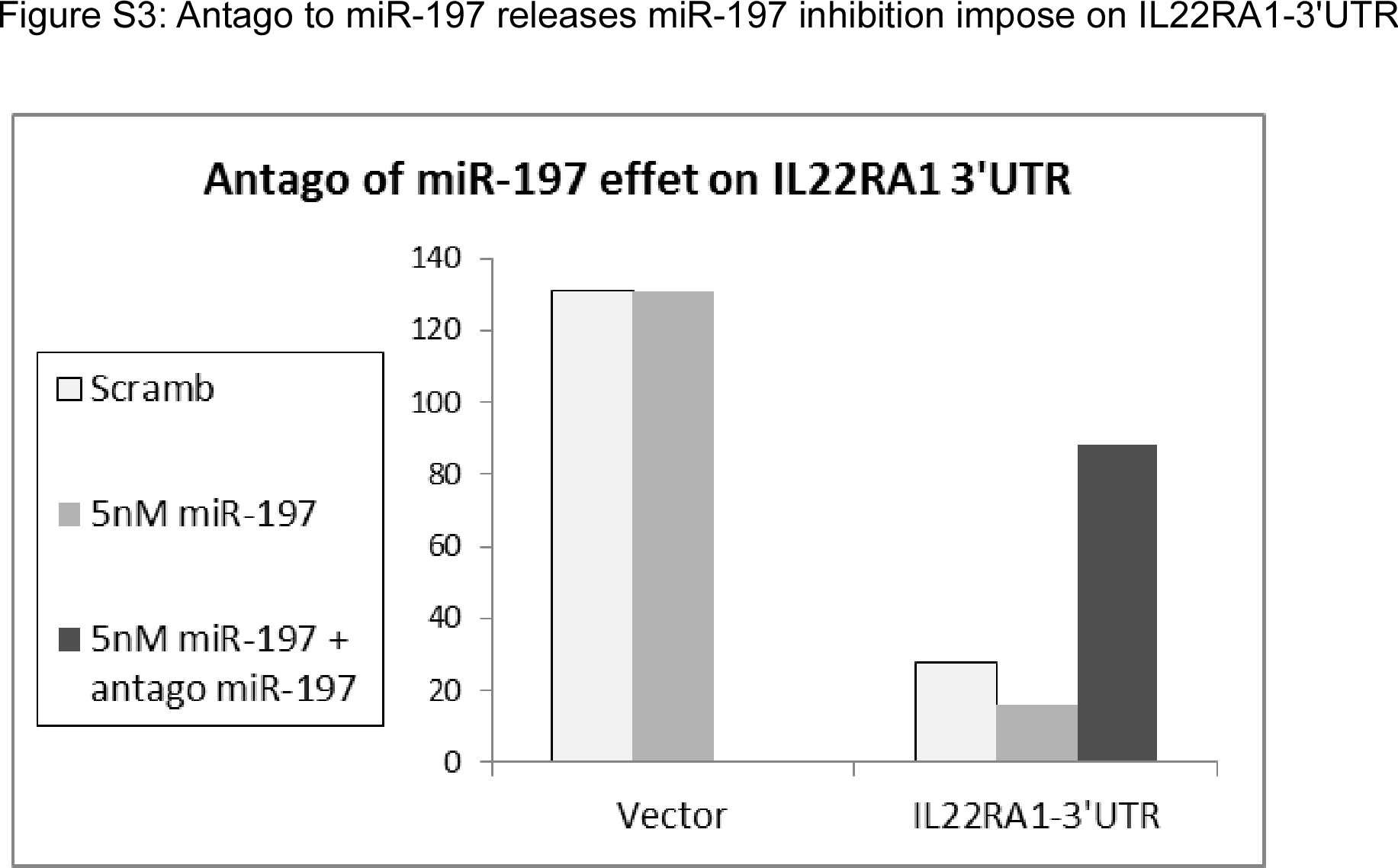

Supplement: Figure S3 — Antago to miR-197 releases miR-197 inhibition impose on IL22RA1-3′UTR depended. HaCaT cells were co-transfected with vector plasmid or luciferase-IL22RA1-3′UTR plasmid and 5 nM of scrambled control RNA, or mimic miR-197 RNA. In parallel one set of cells was transfected with 5 nM of mimic miR-197 together with antago-miR-197. The graph represent and average of at list three independent experiments. (TIF) [file pone.0107467.s003.tif]

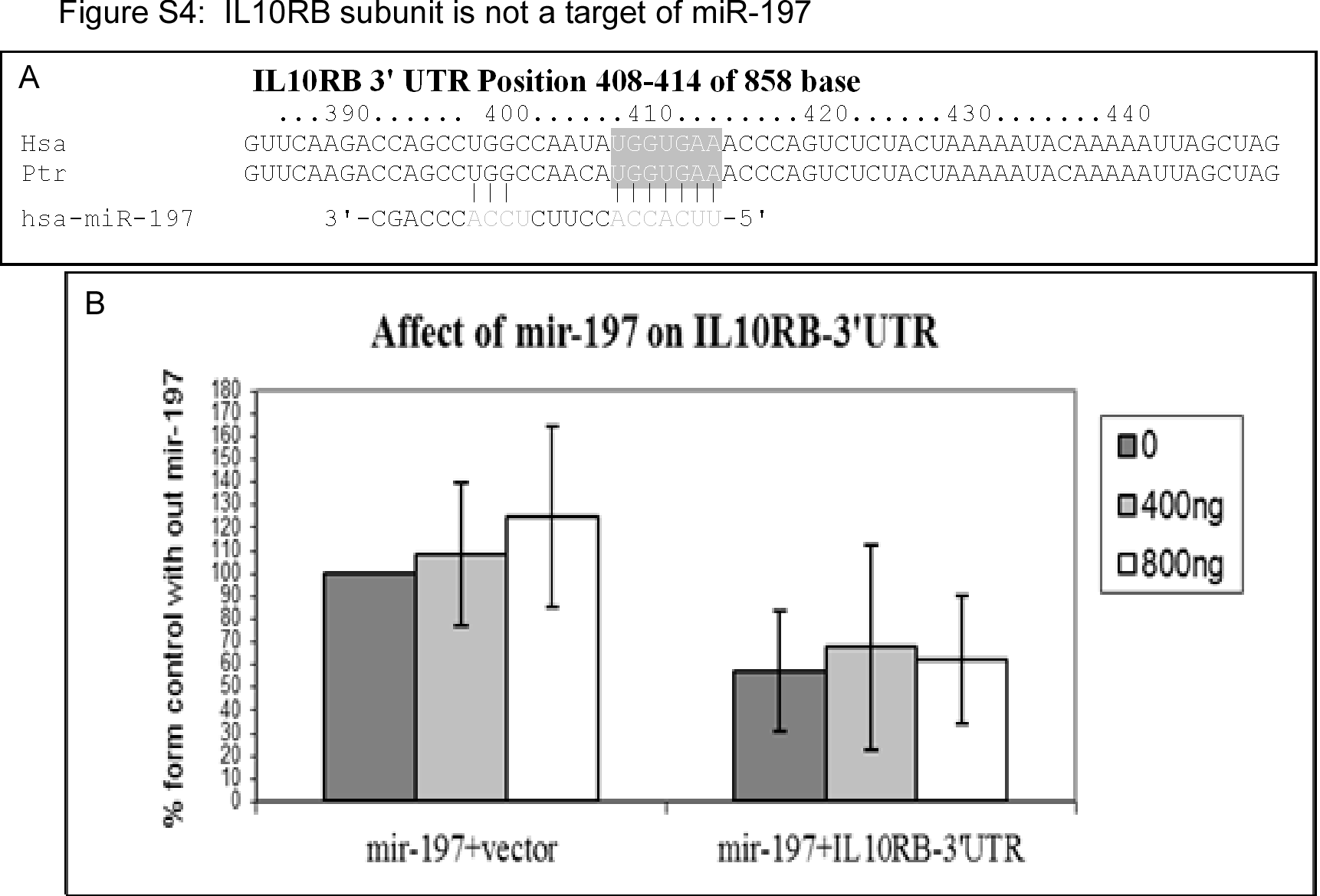

Supplement: Figure S4 — IL10RB subunit is not a target of miR-197. A) MiR-197 biding site in the IL10RB 3′UTR. B) HaCaT cells were co-transfected with vector or IL10RB-3′UTR plasmid with a miR-197 expressing plasmid at different concentrations. In each experiment the same set of plasmids were transfected in triplicates. The graph presents the average of 4 independent experiments. The results of cells transfected with vector lacking the IL10RB-3′UTR and without miR-197 expressing plasmid was valued as 100%. (TIF) [file pone.0107467.s004.tif]

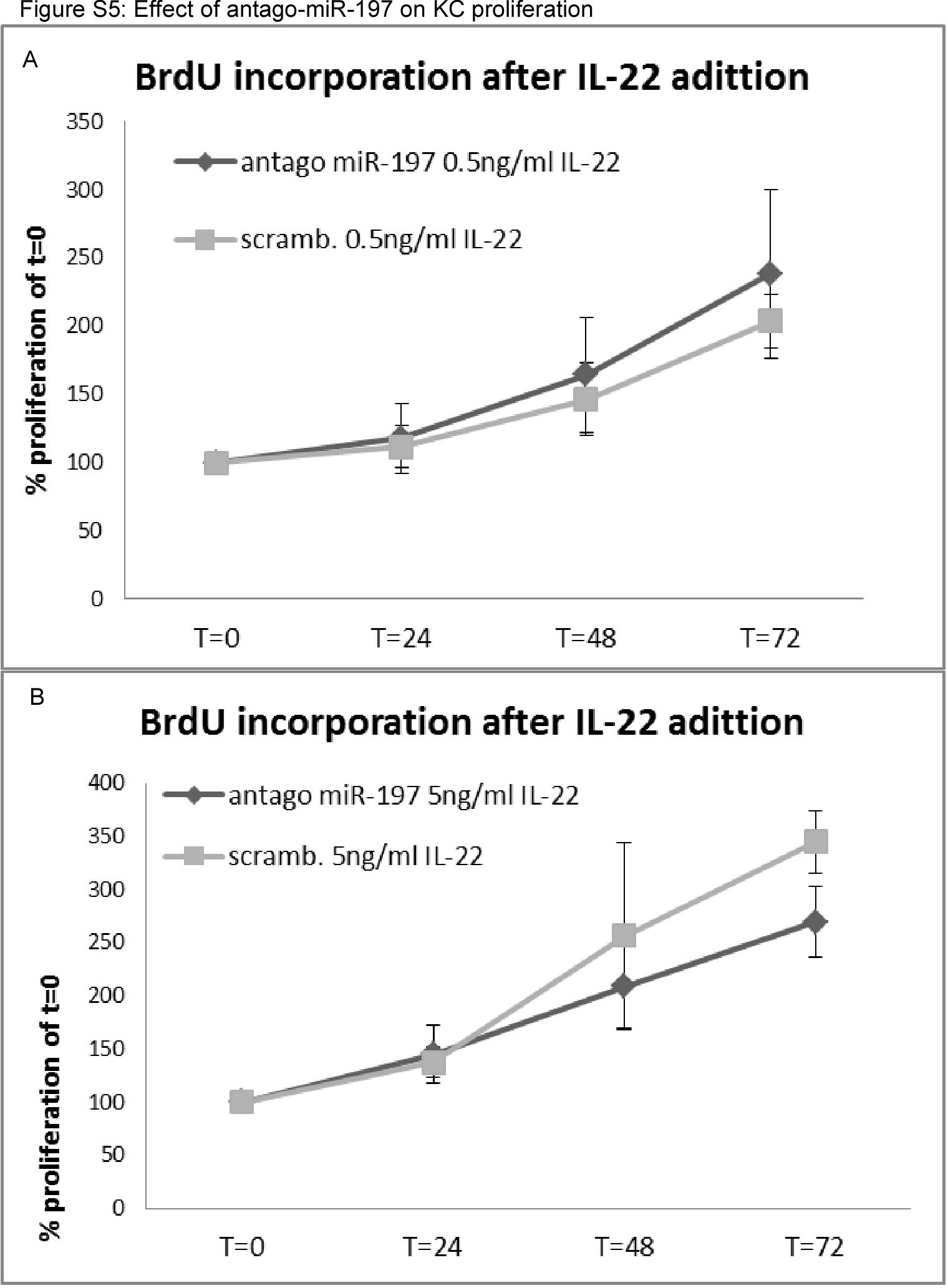

Supplement: Figure S5 — Effect of antago-miR-197 on KC proliferation. HaCaT cells were transfected with antago-miR-197 or scrambled control RNA. 24 h later cells were treated or not with the indicated IL-22 concentrations. Next, BrdU incorporation assay was performed as described in figure 1b. (TIF) [file pone.0107467.s005.tif]
