# Supplementary material for: The Crosstalk between IL-22 Signaling and miR-197 in Human Keratinocytes
Source: PLoS One. 2014 Sep 10;9(9):e107467. doi: 10.1371/journal.pone.0107467 (PMC4160297; doi:10.1371/journal.pone.0107467)
Supplement: Methods S1 — Chromatin Immunoprecipitation (ChIP) Assay. (DOCX) [file pone.0107467.s006.docx]

# Methods S1: Chromatin Immunoprecipitation (ChIP) Assay:

107 primary human keratinocytes were treaded or not with 0.5ng/ml of IL-22 for 30'. Next, formaldehyde (Sigma) was added to final concentration of 1%, for 10'. To quench the cross link glycine to final concentration of 0.125M was added for 5'. Cells were collected and wash twice in PBS, containing protease inhibitors (Complete mini, Roche Applied Science) and Pepstatin (Sigma). Next pellet was washed sequentially for 10 min each in 5 ml of buffers as described in Lysates were sonicated 6 X 10sec bursts to generate DNA fragment ~1000bp. Debris were removed by centrifugation for 10 min at 1000g, at 4^0^C. 15-20µg of DNA from each treatment was first diluted in dilution buffer (0.01% SDS, 1.1% Triton X-100, 1.2 mM EDTA, 16.7 mM Tris–HCl (pH 8.1) and 167 mM NaCl). Lysates were pre-cleared in 40µl of protein A+ salmon sperm beads (Upstate Biotechnology) for 30 min at 4^0^C. Equal amount from each treatment was taken as input control. Equal aliquots of each treatment was subject to immuno precipitation with either control human Ab (IgG) or with Phospho-Stat3 (Tyr705) Antibody (Cell Signaling #9131). After overnight at 4^0^C, 60 µl of protein A+ salmon sperm beads was added, for 2 h at 4^0^C. Next pellets were washed sequentially for 5 min each in 2.5 ml of buffers as described in [66]. The immune complexes were eluted as described in [66]. Next de-crosslinked was performed by adding 16 μl of 5M NaCl, 8 µl of 0.5M EDTA (pH 6.5) and 16 µl of 1M Tris–HCl (pH 8.1) and incubated O.N at 65^0^C. DNA was purified by first, adding Proteinase K (10mg/ml) for 2h at 55^0^C follow by phenol/chloroform (sigma) extraction and ethanol precipitation. Equal aliquots of each sample were analyzed by quantitative real time PCR.

The ChIP qRT of miR-197 promoter was performed with power Syber green with the following primers:

F: 5'AGTGGGTGGTCTTTTACAGCA3' R: 5'GACCTTTTCACCCTGCTTCA3' Tm=60^0^c according to the manufactures protocol (Applied Biosystems Inc Foster City CA 94404).
