# Supplementary material for: The Crosstalk between IL-22 Signaling and miR-197 in Human Keratinocytes
Source: PLoS One. 2014 Sep 10;9(9):e107467. doi: 10.1371/journal.pone.0107467 (PMC4160297; doi:10.1371/journal.pone.0107467)
Supplement: Methods S2 — Migration Assay. (DOCX) [file pone.0107467.s007.docx]

## Methods S2: Migration Assay:

24,000 cells were seeded in platypus 96 wells plate. When 80% confluence was achieved, cells were serum starved for 24 h and only then 0/0.5/5ng per ml IL-22 was added and the platypus stoppers were removed. At this time point one well of every line was fixated and used as a control (T=0). 48 h post IL-22 addition cells were washed twice with PBS and fixated with 70% ethanol for 15 min. Analysis was performed by imageJ. The area without cells was measured and subtracted from the T=0 area, then calculation of migration percentage was determined.
